# Ultra-Processed Food Consumption and Systemic Inflammatory Biomarkers: A Scoping Review

**DOI:** 10.3390/nu17183012

**Published:** 2025-09-20

**Authors:** Jacopo Ciaffi, Luana Mancarella, Claudio Ripamonti, Veronica Brusi, Federica Pignatti, Lucia Lisi, Francesco Ursini

**Affiliations:** 1 Medicine & Rheumatology Unit, IRCCS Istituto Ortopedico Rizzoli, 40136 Bologna, Italy; luana.mancarella@ior.it (L.M.); claudio.ripamonti@ior.it (C.R.); veronica.brusi@ior.it (V.B.); federica.pignatti@ior.it (F.P.); lucia.lisi@ior.it (L.L.); francesco.ursini2@unibo.it (F.U.); 2 Department of Biomedical and Neuromotor Sciences (DIBINEM), Alma Mater Studiorum University of Bologna, 40123 Bologna, Italy

**Keywords:** ultra-processed, food, inflammation, biomarkers, C-reactive protein, interleukins

## Abstract

Background/Objectives: The consumption of ultra-processed foods (UPF) has increased worldwide and has been hypothesized to contribute to chronic diseases, including conditions characterized by inflammatory dysregulation. We conducted a scoping review to map the human evidence on the relationship between UPF consumption and systemic inflammatory biomarkers. Methods: We developed a search strategy combining terms for UPF with terms for circulating inflammatory biomarkers, including C-reactive protein (CRP/hs-CRP), interleukin-6 (IL-6), tumor necrosis factor-α (TNF-α), interleukin-1β (IL-1β), interleukin-8 (IL-8), monocyte chemoattractant protein-1 (MCP-1), plasminogen activator inhibitor-1 (PAI-1), and leptin. Findings were synthesized separately for children/adolescents and adults. Results: A total of 24 studies were included. CRP/hs-CRP was assessed in 21; IL-6 in 9; TNF-α in 8; IL-1β in 5; leptin in 5; MCP-1 in 5; PAI-1 in 5; and IL-8 in 2. In children/adolescents, CRP/hs-CRP tended to be higher with greater UPF intake in large cohorts and in preterm infants, whereas smaller or clinically selected samples did not show an association. For other biomarkers, IL-6 generally did not vary with UPF, TNF-α and IL-1β showed no association across studies, and the two IL-8 analyses yielded mixed results. In adults, 11/17 analyses reported higher CRP/hs-CRP levels with greater UPF intake, 5/17 reported no association, and 1/17 reported an association limited to women. IL-6 was predominantly higher with greater UPF intake; TNF-α likewise tended to be higher with UPF across several settings; IL-1β showed no association; MCP-1 and PAI-1 provided limited, inconsistent signals; leptin results were mixed. Conclusions: Higher UPF consumption is frequently associated with elevated systemic inflammatory biomarkers—most consistently CRP/hs-CRP—across adults and selected pediatric contexts. Signals for IL-6 and TNF-α appear in specific populations, whereas IL-1β, MCP-1, PAI-1, and leptin show inconsistent patterns.

## 1. Introduction

According to the NOVA food classification, ultra-processed foods (UPFs) are industrially manufactured products with minimal whole foods, typically comprising five or more ingredients, including added sugars, oils, fats, salt, and various additives [1]. Typical examples include sugary soft drinks, sweetened cereals, packaged snacks, instant sauces, ready-to-eat meals, and processed meats [2]. The global popularity of UPF is driven by several factors: its shelf stability, little preparation requirements, and strong marketing, rendering it particularly attractive to consumers seeking convenience [3]. Nonetheless, nutritionally, UPFs are energy-dense products, with elevated levels of refined carbohydrates, saturated fats, sodium, and additives, while being deficient in fiber, vitamins, and other critical micronutrients for health [4]. UPF has become increasingly common in contemporary diets, prompting concerns over its influence on chronic diseases [5]. UPF, characterized by hyperpalatability, affordability, and convenience, is now acknowledged as a contributing cause to various chronic disorders [6,7]. In recent decades, the worldwide rise in non-communicable diseases has coincided with a significant increase in UPF consumption [8]. Many of these medical conditions, including metabolic syndrome, hypertension, cardiovascular diseases, type 2 diabetes, inflammatory bowel disease, and demyelinating diseases, are influenced by persistent low-grade inflammation [6,9]. Researchers have suggested that excessive consumption of UPF may induce systemic inflammation, hence hypothesizing an association with the development of non-communicable diseases [10,11,12,13,14].

The poor nutritional quality of UPF and its additive content has been significantly associated with negative health consequences in epidemiological research. Diets heavy in UPF are linked to excessive caloric consumption and high risks of obesity (39% increase), metabolic syndrome (79% increase), and type 2 diabetes (17% increase) [13,15,16]. Elevated intake of UPF has been associated with an increased prevalence of cardiovascular diseases, including coronary artery disease, stroke, and overall mortality [17,18,19]. Moreover, diets heavy in UPF have been associated with an increased prevalence of specific malignancies and neurological or immune-mediated diseases [7,20,21].

Chronic low-grade inflammation is a common factor interconnecting many of these conditions, and dietary patterns rich in UPF are increasingly investigated for their inflammatory potential [22]. Multiple pathways connect UPF to inflammation [6,22]. UPF-heavy diets consist of low nutritional quality and a high concentration of artificial additives and processing-derived substances, which might collectively disrupt gut health and immunological homeostasis [23]. Evidence suggests that both the nutritional composition of UPF and its non-nutritive constituents, along with the impact on the gut flora, contribute to its detrimental inflammatory effects [24]. Several UPFs contain preservatives, emulsifiers, colorants, and other compounds seldom utilized in domestic cooking; these substances may perturb the gut flora, enhance intestinal permeability, and stimulate pro-inflammatory immune responses [25,26]. The result is a pro-inflammatory interior environment (“metaflammation”) that may expedite the onset of chronic diseases [27].

Another potential mechanism is inflammation induced by the nutrients. Excessive intake of saturated fat can modify the gut microbiota towards a more inflammatory state and directly increase circulating pro-inflammatory cytokines and endotoxin concentrations [28]. The insufficient amount of dietary fiber in ultra-processed meals leads to reduced production of anti-inflammatory short-chain fatty acids by gut microbiota, hence compromising intestinal immunological equilibrium [29].

Current evidence suggests a plausible link between UPF, chronic inflammation, and diseases, but additional clarification is necessary [6,22]. To our knowledge, no prior review has systematically mapped the full spectrum of inflammatory biomarkers in relation to UPF consumption in humans. Considering the rapid rise in the consumption of UPF and its effects on metabolic and inflammatory pathways, we decided to conduct a comprehensive evaluation of the impact of UPF on inflammation. This scoping review aims to synthesize available evidence on the association between UPF consumption and inflammatory markers, using findings from studies on the general population and research involving patients with inflammation-related diseases. By analyzing epidemiological data and biological mechanisms, we seek to clarify the extent to which UPFs may affect inflammatory processes and to identify areas for future research.

## 2. Materials and Methods

This scoping review was conducted in accordance with the methodological guidance of the Joanna Briggs Institute [30]. Reporting followed the Preferred Reporting Items for Systematic Reviews and Meta-Analysis extension for Scoping Reviews (PRISMA-ScR) checklist (Supplementary File) [31]. The protocol was registered in the Open Science Framework (OSF) Registries (https://doi.org/10.17605/OSF.IO/W8K2G) on 13 August 2025. Consistent with the process of scoping reviews, no critical appraisal or assessment of bias risk for the included studies was conducted [30].

### 2.1. Eligibility Criteria and Study Selection

The Population, Concept, and Context (PCC) framework was applied to define the research question and eligibility criteria:

- **Population:** we included studies involving humans of any age. Results were considered separately for adults and for children/adolescents.

- **Concept:** eligible studies were those that directly evaluated the association between UPF consumption and systemic inflammatory markers. We included observational or epidemiological studies (e.g., cross-sectional, case–control, cohort) as well as mechanistic human studies (e.g., short-term dietary interventions) only if they reported results on inflammatory markers across different levels of UPF intake (e.g., categories or quantiles of UPF consumption, or continuous associations). To be eligible, studies had to provide a quantitative measure of the relationship (e.g., *p*-value, β coefficient, odds ratio, or mean differences) for at least one systemic inflammatory marker.

The following inflammatory markers were evaluated: C-reactive protein (CRP), high-sensitivity CRP (hs-CRP), erythrocyte sedimentation rate (ESR), fibrinogen, interleukin-6 (IL-6), interleukin-1β (IL-1β), tumor necrosis factor-alpha (TNF-α), interleukin-8 (IL-8), chemokine CCL2/monocyte chemoattractant protein-1 (MCP-1), plasminogen activator inhibitor-1 (PAI-1) and leptin. Studies that reported solely surrogate indices of dietary inflammatory potential, such as the dietary inflammatory index without direct biomarker assessment, were excluded.

- **Context:** studies were eligible regardless of setting, geographical region, or healthcare system. Both general population samples and patient cohorts were included.

We considered randomized controlled trials (RCTs), quasi-experimental studies, prospective and retrospective cohort studies, case–control studies, cross-sectional studies, and qualitative reports. Conference abstracts and proceedings were also eligible.

### 2.2. Search Strategy

We carried out a comprehensive literature search in Medline (via PubMed), Web of Science (WOS), and Embase, on 17 August 2025.

The search strategy combined terms related to dietary exposures (e.g., “ultra-processed food,” “ultra processed food,” “ultraprocessed food,” “ultra-processed foods,” “ultra processed foods,” “ultraprocessed foods”) with those referring to systemic inflammatory markers. Specifically, we included CRP, hs-CRP, ESR, fibrinogen, IL-6, IL-1β, TNF-α, IL-8, CCL2/MCP-1, PAI-1 and leptin.

To enhance comprehensiveness, we conducted manual searches employing pertinent keywords and examined the reference lists of included research to identify additional eligible papers. The development and execution of the search strategy were conducted independently by two investigators (J.C. and L.M.) under the guidance of a senior reviewer (F.U.). No time limits were imposed on the search.

The complete search strings for each database are available in the Supplementary Materials to guarantee transparency and reproducibility.

### 2.3. Study Selection and Data Charting

Following the removal of duplicate records, two reviewers (J.C. and L.M.) independently evaluated all titles and abstracts of the obtained publications. A comprehensive evaluation was subsequently conducted for all studies considered potentially eligible. Disagreements throughout the selection process were addressed by discussion, with a senior investigator (F.U.) consulted where consensus was unattainable.

For each study included, the following data were extracted using a standardized collection form: first author, year of publication, country, study design, sample size, study population, method of exposure assessment for UPF consumption, inflammatory markers evaluated, and main findings concerning the relationship between UPF intake and systemic inflammation.

### 2.4. Synthesis of Results

We conducted a narrative synthesis, organizing findings by population and, within each population, by biomarker. For each biomarker, we prioritize adjusted estimates, report the direction and statistical significance of associations, and note sex-stratified or sensitivity analyses when available. Where a study includes both adolescents and adults, subgroup findings are summarized in both population sections. CRP and hs-CRP are considered jointly a priori.

**UPF consumption and systemic inflammatory biomarkers in children and adolescents**

- This section summarizes studies assessing the association between UPF consumption and systemic inflammatory biomarkers in pediatric and adolescent populations, presented per biomarker.

**UPF consumption and systemic inflammatory biomarkers in adults**

- This section collects evidence from research assessing the influence of UPF consumption on systemic inflammatory markers in adult populations, with results presented per biomarker.

**Summary tables**

- Descriptive tables report study characteristics (author, year, country, design, population, sample size), UPF exposure assessment, biomarkers assessed, and key findings to support the narrative synthesis, and summary tables visually highlight the presence of association between UPF intake and each inflammatory biomarker in the included studies.

## 3. Results

**Figure 1:**
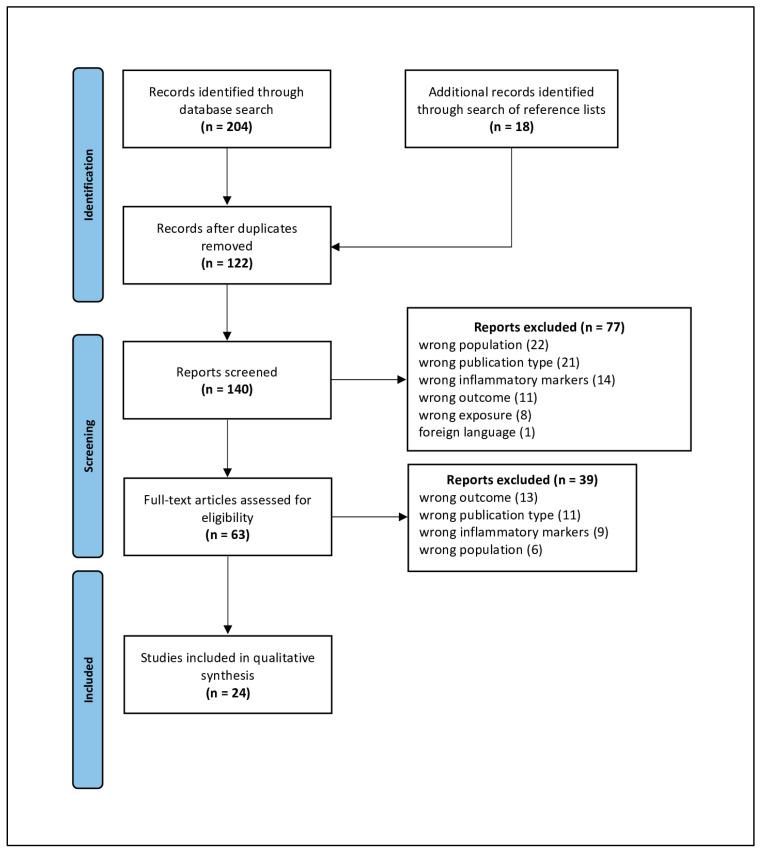
PRISMA 2020 flow diagram. Adapted From: Page, M.J., McKenzie, J.E., Bossuyt, P.M., Boutron, I., Hoffmann, T.C., Mulrow, C.D. et al. The PRISMA 2020 statement: an updated guideline for reporting systematic reviews [32].

The database search yielded 204 records (PubMed: 51; Web of Science: 81; Embase: 72). An additional 18 studies were identified through reference screening. After removal of duplicates, 140 unique records remained. Following title/abstract screening and full-text assessment against eligibility criteria, 24 studies met inclusion and were retained for qualitative synthesis, comprising 23 full papers and 1 conference abstract. A PRISMA flow diagram of the selection process is provided in Figure 1. The included studies were published between 2019 and 2025 and originated from Brazil (6), United States (U.S.) (5), Spain (3), Iran (3), Canada (2), and one each from Norway, Republic of Korea, France, Australia, and Ireland. By population, 5 studies enrolled children or adolescents, 17 enrolled adults, and 2 included both adolescents and adults. Nineteen studies sampled the general population, while 5 focused on specific clinical groups (metabolic syndrome, survivors of childhood acute lymphoblastic leukemia, celiac disease in children, preterm infants, and adults with colon cancer). By design, 19 were cross-sectional, 3 prospective, 1 retrospective, and 1 RCT. Overall, CRP/hs-CRP was assessed in 21/24 studies; IL-6 in 9/24; TNF-α in 8/24; IL-1β in 5/24; leptin in 5/24; MCP-1 in 5/24; PAI-1 in 5/24; and IL-8 in 2/24. ESR and fibrinogen were not assessed in any study; IL-8 was not assessed in adults, and PAI-1 was not assessed in pediatric populations. All study-level details are reported in Table 1, while Table 2 and Table 3 present visual summaries of UPF–biomarker associations for children/adolescents and adults, respectively, with checks (**✓**) for associations and crosses (**X**) for no association.

### 3.1. UPF Consumption and Systemic Inflammatory Biomarkers in Children and Adolescents

#### 3.1.1. UPF and CRP/hs-CRP

Six studies investigated the relationship between UPF intake and CRP or hs-CRP concentrations in pediatric populations (Table 2) [35,38,46,51,52,56].

In the large nationwide ERICA cohort of Brazilian adolescents (n = 6316), high UPF consumption was modestly associated with a greater prevalence of elevated CRP levels, suggesting low-grade systemic inflammation [38]. Similarly, in a cross-sectional study of 391 adolescents in Northeastern Brazil, higher UPF intake was associated with increased hs-CRP concentrations [46]. Among younger children, a Brazilian study including preterm and term infants (n = 90) found that higher UPF intake was independently associated with higher CRP levels, with a significant interaction between prematurity and UPF exposure [52].

By contrast, in a cohort of 151 Brazilian schoolchildren, UPF consumption was linked to adverse lipid and liver profiles, but no consistent associations were observed with hs-CRP [51]. Similarly, cross-sectional analysis including 806 American adolescents reported no association between UPF intake and hs-CRP [56]. Finally, in Canadian survivors of childhood acute lymphoblastic leukemia (n = 241; including 85 under 18 years), diet quality indices and UPF consumption were associated with dyslipidemia and insulin resistance, but no significant associations emerged with CRP [35].

Overall, across the six pediatric studies, three reported higher CRP/hs-CRP with greater UPF intake, whereas three found no association, indicating signals concentrated in large adolescent cohorts and preterm infants while smaller or clinically selected samples more often showed no association.

#### 3.1.2. UPF and IL-6

Four pediatric studies evaluated the association between UPF consumption and circulating IL-6 (Table 2) [35,46,48,51]. In Canadian survivors of childhood acute lymphoblastic leukemia (n = 241; including 85 under 18 years), stratified models by obesity status showed higher IL-6 at greater UPF intake in both obese and non-obese participants [35].

By contrast, in a population-based study of Brazilian adolescents (n = 391), higher UPF consumption was not associated with elevated IL-6 levels [46]. Consistent findings emerged in Spanish children with celiac disease (n = 53) compared to healthy controls (n = 32), where greater UPF intake was not linked to increased IL-6 concentrations [48]. Finally, in Brazilian schoolchildren (n = 151), higher UPF consumption was also not associated with higher IL-6 values in multivariable analyses [51].

Overall, pediatric evidence does not show a consistent association between UPF and IL-6; the only positive signal appears among childhood cancer survivors but requires confirmation.

#### 3.1.3. UPF and TNF-α

Four studies investigated the relationship between UPF intake and TNF-α levels in pediatric populations, and all had negative results (Table 2) [35,46,48,51]. In Canadian survivors of childhood acute lymphoblastic leukemia (n = 241; including 85 under 18 years), no significant associations were observed between UPF consumption and TNF-α concentrations [35]. In a population-based sample of Brazilian adolescents (n = 391), higher UPF was related to higher hs-CRP and leptin, but TNF-α was not associated [46]. Similarly, among Spanish children with celiac disease (53 cases) and healthy controls (n = 32), TNF-α concentrations did not differ across UPF intake categories or versus controls [48]. Finally, in Brazilian schoolchildren (n = 151), UPF intake related to lipid and liver enzymes but not to TNF-α [51].

Overall, the pediatric evidence indicates no association between UPF consumption and TNF-α.

#### 3.1.4. UPF and IL-1β

Only one pediatric study examined IL-1β in relation to UPF consumption (Table 2). In Spanish children with celiac disease (n = 53) and healthy controls (n = 32), higher UPF intake was not associated with increased IL-1β concentrations [48].

The evidence in pediatric populations is very limited and does not support an association between UPF intake and higher IL-1β; confirmation in larger and more diverse cohorts is needed.

#### 3.1.5. UPF and IL-8

Evidence for IL-8 derives from two pediatric studies (Table 2) [46,48]. In a population-based sample of Brazilian adolescents (n = 391), those in the highest UPF tertile showed significantly higher IL-8 concentrations than the lowest tertile in adjusted models [46]. In contrast, among Spanish children with celiac disease (n = 53) and healthy controls (n = 32), IL-8 concentrations did not differ across UPF intake categories after adjustment [48].

Overall, pediatric evidence on IL-8 is limited and mixed, suggesting a positive association in one adolescent cohort but no differences in a disease-specific sample, warranting confirmation in additional populations.

#### 3.1.6. UPF and MCP-1

Among the pediatric studies, MCP-1 was evaluated only in Spanish children with celiac disease (n = 53) and healthy controls (n = 32) by comparing celiac participants with <50% vs. ≥50% of energy from UPF (plus controls) (Table 2) [48]. MCP-1 concentrations did not differ significantly across UPF intake categories, indicating no clear association between UPF consumption and MCP-1 in this sample.

Overall, pediatric evidence on MCP-1 is very limited and, in the only available study, does not support a relationship between UPF intake and MCP-1.

#### 3.1.7. UPF and Leptin

Two studies investigated the association between UPF intake and leptin levels in pediatric populations (Table 2) [35,46]. In Canadian survivors of childhood acute lymphoblastic leukemia (n = 241; including 85 under 18 years), diet quality indices and UPF consumption were associated with several cardiometabolic outcomes, but no significant associations were found with leptin concentrations [35]. Conversely, in a population-based study of Brazilian adolescents (n = 391), those in the highest UPF consumption tertile showed significantly higher leptin levels compared to peers with lower intake [46].

Overall, current pediatric evidence suggests inconsistent findings, with a positive association between UPF consumption and leptin reported in a general adolescent cohort, but not among childhood cancer survivors.

### 3.2. UPF Consumption and Systemic Inflammatory Biomarkers in Adults

#### 3.2.1. UPF and CRP/hs-CRP

Seventeen studies examined UPF in relation to CRP/hs-CRP in adults (Table 3) [33,34,35,36,37,39,40,41,42,43,44,45,47,53,54,55,56]. Of these, 11/17 reported a positive association, 5/17 described no association, and 1/17 showed a sex-specific pattern (positive in women, absent in men). Large general-population studies, such as those by Baric et al. (Canada; n = 6517), Kityo et al. (U.K. Biobank; n = 72,817), Lane et al. (Australia; n = 2018), Millar et al. (Ireland; n = 1986), and Zhao et al. (U.K. Biobank; n = 173,889 and NHANES cohort; n = 2734), reported correlation between greater UPF intake and higher CRP/hs-CRP [34,43,44,47,55,56].

Positive associations also emerged in specific contexts: pregnancy cohorts in the U.S. (n = 350) and Norway (n = 2984), colon cancer patients in the U.S. (n = 796), and regional samples of obese and overweight women in Iran (n = 391 and n = 285) [33,37,41,42,54].

In contrast, absence of association between UPF consumption and CRP/hs-CRP was reported in a Canadian sample of leukemia survivors (n = 241; including 156 above 18 years), a Spanish outpatient sample (n = 152), another Iranian cohort (n = 221), a short randomized crossover feeding trial from the U.S. (n = 20), and a French cohort (n = 1594) [35,36,39,40,53].

A sex-specific pattern was observed by Lopes et al. in a Brazilian study on civil servants from public universities/research institutions (n = 8468), where higher UPF consumption was related to higher CRP in women but not in men after sociodemographic adjustment.

Overall, adult studies generally show an association between greater UPF intake and higher CRP/hs-CRP across large population cohorts and several clinical settings, alongside variability across study designs, including absence of association in short feeding trials, and a single cohort reporting a female-specific association.

#### 3.2.2. UPF and IL-6

Six studies evaluated UPF in relation to circulating IL-6 in adults (Table 3) [35,37,47,49,50,54]. Of these, 5/6 reported a positive association and 1/6 found no association.

Higher IL-6 with greater UPF intake was observed in Canadian long-term leukemia survivors (n = 241; including 156 above 18 years), Spanish older adults with metabolic syndrome (n = 92), young-adult cohorts from Portugal and Brazil (n = 524 and n = 2888, with a sex-specific pattern: the association is present in women in Portugal and in men in Brazil), an Irish primary-care sample (n = 1986), and colon cancer patients from the U.S. (n = 796) [35,47,49,50,54]. In contrast, no association was detected in an American pregnancy cohort (n = 350) [37].

Overall, adult IL-6 evidence points to a prevailing positive association between higher UPF intake and higher IL-6 across diverse settings, with sex-specific patterns in young adults and a single negative finding in pregnancy.

#### 3.2.3. UPF and TNF-α

Five studies assessed UPF in relation to circulating TNF-α in adults; three reported a positive association and two reported no association (Table 3) [35,37,47,49,54].

Higher TNF-α with greater UPF intake was observed in Spanish older adults with metabolic syndrome (n = 92), U.S. colon cancer patients (n = 796), and an Irish primary-care sample (n = 1986) [47,49,54].

In contrast, no association was detected in a U.S. pregnancy cohort (n = 350) or Canadian long-term leukemia survivors (n = 241; including 156 above 18 years) [35,37].

Overall, adult TNF-α evidence points toward a prevailing positive association between higher UPF intake and higher TNF-α, including in primary-care and disease-specific settings, while in pregnancy and long-term cancer survivor cohorts no association was found.

#### 3.2.4. UPF and IL-1β

Four studies assessed UPF in relation to circulating IL-1β in adults, and all four reported no association (Table 3) [33,39,41,49]. This included three cohorts of overweight/obese adult women in Iran (n = 285, n = 221, n = 391) and a cohort of older adults with metabolic syndrome in Spain (n = 92) [33,39,41,49].

Overall, available adult evidence does not support an association between UPF consumption and IL-1β levels.

#### 3.2.5. UPF and MCP-1

Four studies assessed UPF in relation to circulating MCP-1 in adults; three reported no association and one reported a borderline positive association (Table 3) [33,39,41,49].

No association was observed in two samples of overweight/obese women in Iran (n = 285 and n = 391) and in older adults with metabolic syndrome in Spain (n = 92), whereas a third Iranian cohort of overweight/obese women (n = 221) reported a borderline increase in MCP-1 with higher UPF intake [33,39,41,49].

Overall, adult evidence for MCP-1 is limited and does not consistently support an association with UPF consumption.

#### 3.2.6. UPF and PAI-1

Five studies assessed UPF in relation to circulating PAI-1 in adults; one reported a positive association and four reported no association (Table 3) [33,39,40,41,47]. A positive relationship was observed in overweight/obese women in Iran (n = 221) [39].

No association was found in two additional cohorts of overweight/obese women in Iran (n = 285 and n = 391), in a short, randomized crossover feeding trial conducted in a metabolic ward in the U.S. (n = 20), or in a primary-care adult sample in Ireland (n = 1986) [33,40,41,47].

Overall, adult evidence for PAI-1 is limited, with a single positive report and four analyses finding no association.

#### 3.2.7. UPF and Leptin

Four studies assessed UPF in relation to circulating leptin in adults; two reported a positive association and two reported no association (Table 3) [35,40,47,49].

Higher leptin with greater UPF intake was observed in older adults with metabolic syndrome in Spain (n = 92) and in a primary-care adult sample in Ireland (n = 1986) [47,49].

In contrast, no association was detected in Canadian long-term leukemia survivors (n = 241; including 156 above 18 years) or in a short, randomized crossover feeding trial conducted in a metabolic ward from the U.S. (n = 20) [35,40].

Overall, adult evidence for leptin is mixed, with signals in metabolic-risk and primary-care cohorts, while cancer-survivor and short feeding-trial settings report no association.

## 4. Discussion

UPF accounts for an increasing proportion of dietary intake worldwide and has been associated with several chronic diseases characterized by low-grade systemic inflammation [6]. In this context, we conducted a scoping review to summarize human evidence regarding the association between UPF consumption and circulating inflammatory biomarkers across various age groups, examining patterns by biomarker and population while identifying methodological sources of heterogeneity that may explain inconsistent findings.

We included 24 studies; all were published between 2019 and 2025. CRP/hs-CRP emerged as the most consistently associated biomarker, particularly in adults. For cytokines, IL-6 and TNF-α generally showed positive associations with UPF intake, whereas IL-1β, MCP-1, PAI-1, and leptin yielded less consistent evidence. Notably, ESR and fibrinogen were not evaluated, and IL-8 was examined only in pediatric populations. In children and adolescents, larger cohorts of teenagers and preterm-infant samples suggested higher CRP/hs-CRP with greater UPF intake, while smaller or clinically selected samples more often did not show an association. For pediatric markers beyond CRP/hs-CRP, evidence was limited: IL-6 generally did not vary with UPF, TNF-α showed no association across studies, IL-1β showed no association in the single study available, and IL-8 evidence was limited and mixed across two studies.

Overall, the adult evidence aligns higher UPF intake with higher CRP/hs-CRP, with more variability for cytokines and other markers. This pattern is biologically plausible. Diets high in UPF are typically energy-dense, abundant in added sugars and saturated fats, and deficient in fiber and micronutrients—characteristics consistently associated with elevated inflammatory biomarkers in children, adolescents, and adults [57].

In a nationally representative investigation from the U.S., UPF contributed approximately 58% of total energy and almost 90% of added sugars; each 5% increase in energy from UPF was associated with a 1% increase in energy from added sugars, exhibiting a nearly linear gradient across UPF quintiles [58]. Added sugars, refined starches, and high-glycaemic loads can exacerbate postprandial glycaemia and lipaemia, oxidative stress, and subsequent cytokine responses, potentially increasing CRP/hs-CRP and IL-6 levels [58,59].

Saturated fats may further contribute: a systematic review revealed a positive correlation between saturated fatty acids (SFA) and IL-6 as well as endothelial activation markers such as soluble intercellular adhesion molecule-1 (sICAM-1), whereas data regarding hs-CRP were inconsistent and there was little support for the relationship between SFA and adipokines [60].

Additionally, dietary-pattern research suggests that the diet–inflammation relationship is partially mediated by adiposity: higher-quality dietary patterns correlate with reduced CRP and leptin levels, while “Western” dietary patterns are associated with higher levels, but this correlation attenuates after adjusting for BMI [61,62]. Adiposity, particularly central or visceral fat, is strongly related to hs-CRP through inflammation in adipose tissue and hepatic acute-phase responses [63]. The evidence substantiates a mediation pathway (UPF → adiposity → inflammation) that aligns with the attenuation observed in several studies included in our review after comprehensive covariate adjustment.

Importantly, biomarkers such as CRP, IL-6, TNF-α, and leptin are frequently elevated in individuals with overweight or obesity independent of dietary exposures [64,65,66]. Accordingly, the associations observed in included studies should not be interpreted as solely attributable to UPF intake, but rather as the result of interactions between adiposity, metabolic status, and dietary patterns.

However, nutrient content and adiposity are unlikely to be the whole story. A large cross-sectional study from the Moli-sani cohort linked increased consumption of UPF with an elevated composite inflammatory score (hs-CRP, white blood cells, platelets, granulocyte–lymphocyte ratio) [67]. When the energy-adjusted Dietary Inflammatory Index (E-DII) into the models, the relationship with processed foods diminished significantly (~88% attenuation), but the association with UPF exhibited only a ~33% attenuation, indicating a substantial non-nutritional component unique to ultra-processing [67].

Proposed mechanisms encompass additives (e.g., emulsifiers, non-nutritive sweeteners), compounds derived from processing (e.g., acrylamide, advanced glycation end-products), and contaminants from packaging (e.g., bisphenols), which exert effects through microbiota disturbance, barrier impairment, endotoxemia, and activation of the innate immune system [67,68].

Prospective data further reinforce both the epidemiologic signal and mechanistic plausibility. In the Malmö Diet and Cancer Study, increased consumption of UPF was associated with elevated risks of cardiovascular disease outcomes [69]. Substitution analyses indicated that replacing UPF with unprocessed or minimally processed foods could reduce these risks [69]. Proteomic analysis in a subcohort revealed that higher levels of inflammatory and endothelial-related proteins (e.g., IL-18, TNF-R1/2, CSF-1, thrombomodulin, resistin) correlated with higher UPF intake, many of which were prospectively linked to cardiovascular disease [69].

In individuals with type 2 diabetes, increased consumption of UPF predicted cardiovascular outcomes, with a detectable mediation by CRP and BMI, among other variables [70]. A similar analysis described higher risks of microvascular complications, particularly diabetic kidney disease, again mediated by BMI and CRP [71]. Beyond cardiometabolic disease, increased intake of UPF was associated with to the onset of rheumatoid arthritis in the U.K. Biobank, with partial mediation through CRP, high-density lipoprotein cholesterol, white blood cell count, and composite inflammatory indices [72]. These studies collectively suggest that systemic inflammation is integral to the UPF–disease relationship and that multiple, partially overlapping pathways—nutrient-driven, adiposity-related, and processing-specific—likely operate synergistically.

Two additional considerations are relevant when comparing studies. First, classification choices for UPF can materially affect results. In NHANES 2015–2018, the CRP association was evident in adjusted models only when UPF definitions excluded products with high whole-grain content; the standard definition diluted the signal [73]. This highlights intra-category variability and the necessity of describing UPF subsets (e.g., beverages, sweets, savory snacks) individually when feasible. Second, design and measurement matter: most studies were cross-sectional with single-time-point biomarkers; the only short metabolic-ward trial did not detect hs-CRP differences over two weeks [40]. Small quasi-experimental studies that reduce UPF by enhancing plant-food variety over a period of weeks similarly demonstrate minimal changes in biomarkers, perhaps due to the brief duration, small sample sizes, and inadequate assays [74]. These patterns argue for longer interventions with repeated measures and panels beyond CRP alone.

The mediation results align with clinical observations from nutritional interventions [67,70,72]. In very-low-calorie ketogenic diet (VLCKD) trials, significant clinical improvement was observed in knee osteoarthritis associated with weight reduction, consistent with adiposity-mediated advantages, while in fibromyalgia, improvements were not statistically attributable to changes in BMI, indicating partially weight-independent mechanisms [75,76]. Mechanistically, ketosis and β-hydroxybutyrate inhibit NLRP3 inflammasome signaling and modulate cytokines, illustrating that dietary exposures can influence inflammation both through and beyond adiposity [77]. While these interventions are not specifically aimed at reducing UPF, they support a nuanced mediation paradigm where adiposity, nutrient quality (e.g., sugars/saturated fatty acids), and non-nutritional characteristics of ultra-processing collectively influence systemic inflammation.

The strengths of our scoping review encompass the extensive coverage of inflammatory biomarkers, parallel synthesis in pediatric and adult cohorts, clear presentation of counts by biomarker, and the utilization of summary matrices to enable quick comparisons.

Limitations reflect those of the underlying literature: predominance of cross-sectional designs; variability in UPF exposure metrics and in the measurement of CRP versus hs-CRP and cytokines; limited amount of prospective and experimental evidence; and potential misclassification of processing categories. IL-8 was evaluated in pediatric population only, while PAI-1 was analyzed solely in adults; ESR and fibrinogen were not assessed in the studies included, hence limiting the possibility of inference. The absence of consistent association between UPF consumption and biomarkers such as IL-1β, MCP-1, PAI-1, and leptin, may reflect methodological limitations or population differences but could also indicate a genuine lack of correlation. Further research is needed to determine whether these negative or inconsistent findings represent true absence of effects or context-dependent variations. Finally, residual confounding may persist despite multivariable adjustment in several studies, and RCTs with standardized dietary interventions in healthy individuals would be needed to establish whether UPF directly alters systemic inflammatory biomarkers.

Considering these limitations, it is also important to consider future directions. Our view is that reducing the consumption of UPF should be regarded as a public health priority. Convenience, affordability, and aggressive marketing largely drive the widespread use of UPF, but regulatory and policy measures are needed to counteract these factors and promote healthier alternatives. Solid evidence already demonstrates that high UPF intake increases the risk of metabolic syndrome, cardiovascular disease, cancer, and musculoskeletal conditions [7,20,78]. By showing consistent associations with systemic inflammation, our review reinforces the notion that UPF is detrimental to health across multiple domains and should be limited as much as possible.

## 5. Conclusions

Human evidence—most consistently for CRP/hs-CRP in adults—supports an association between greater UPF consumption and higher levels of systemic inflammatory biomarkers, with additional signals for IL-6 and TNF-α in selected contexts and mixed findings for other markers. Differences in UPF classification, exposure metrics, populations, and biomarker panels likely explain part of the heterogeneity. Future work should prioritize standardized UPF metrics, repeated biomarker measures, explicit mediation analyses (adiposity, diet quality, and processing-specific factors), and adequately powered prospective studies and longer interventions. Such designs will clarify causality, identify modifiers, and better define the clinical significance of UPF-related inflammatory changes.

## Figures and Tables

**Table 1:** Characteristics of the Included Studies.

First Author	Year of Publication	Country of Affiliation of First Author	Publication Type	Study Design	Sample Size	Study Population	Exposure Assessment	Inflammatory Markers Assessed	Key Findings
Bahrampour N. [33]	2022	Iran	Full-text article	Cross-sectional	285	Overweight and obese adult women	UPF % of total dietary intake	hs-CRP, IL-1β, MCP-1, PAI-1	Higher UPF consumption was associated with increased hs-CRP levels, while no significant associations were observed for MCP-1, PAI-1, or IL-1β.
Baric L. [34]	2025	Canada	Full-text article	Cross-sectional	6517	Adults from the Canadian Health Measures Survey	UPF % of total dietary intake	CRP	Higher UPF consumption was associated with increased CRP levels.
Bérard A. [35]	2020	Canada	Full-text article	Cross-sectional	241 (156 adults; 85 <18 years)	Long-term survivors of childhood acute lymphoblastic leukemia	UPF % of total dietary intake	CRP, IL-6, TNF-α, leptin	Higher UPF consumption was associated with increased IL-6 levels in both obese and non-obese participants. A non-significant trend was observed for higher TNF-α. No associations were found for CRP or leptin.
Contreras-Rodriguez O. [36]	2023	Spain	Full-text article	Cross-sectional	152	Adults consulting for weight loss	UPF % of total dietary intake	hs-CRP	UPF consumption was not associated with hs-CRP.
Cummings J. [37]	2022	U.S.	Full-text article	Prospective cohort	458 enrolled; biomarker subsample n = 350	Pregnant women from a metropolitan area in North Carolina	UPF % of total energy intake	CRP, IL-6, TNF-α	Higher UPF consumption during pregnancy was associated with increased CRP, while associations with IL-6 and TNF-α were not significant.
Dos Santos G.R. [38]	2025	Brazil	Full-text article	Cross-sectional	6316	Adolescents	UPF % of total energy intake	CRP	Higher total UPF consumption was modestly associated with a higher prevalence of elevated CRP.
Hajmir M. [39]	2023	Iran	Full-text article	Cross-sectional	221	Overweight and obese adult women	UPF intake categorized by quartiles	hs-CRP, IL-1β, MCP-1, PAI-1	UPF quartiles were significantly associated with PAI-1, marginally associated with MCP-1, and not associated with hs-CRP or IL-1β after adjustment.
Hall K.D. [40]	2019	U.S.	Full-text article	Randomized controlled trial	20	Adults hospitalized in metabolic ward, undergoing two 2-week periods: UPF diet vs. unprocessed diet, ad libitum intake.	UPF-based diet vs. unprocessed diet, in crossover design	hs-CRP, PAI-1, leptin	No statistically significant differences were observed between the ultra-processed and unprocessed diets in hs-CRP, leptin or PAI-1 levels, although hs-CRP tended to be lower after the unprocessed diet compared with baseline.
Hosseininasab D. [41]	2022	Iran	Full-text article	Cross-sectional	391	Overweight and obese adult women	UPF intake categorized by quartiles	hs-CRP, IL-1β, MCP-1, PAI-1	Higher UPF consumption was associated with increased hs-CRP levels in tertile-based analyses, but no association was observed for IL-1β, MCP-1 and PAI-1.
Kelsey P.T. [42]	2022	Norway	Full-text article	Cross-sectional	2984	Nationally recruited pregnant women	UPF % of total energy intake	CRP	Higher UPF intake was weakly associated with higher CRP.
Kityo A. [43]	2025	Republic of Korea	Full-text article	Cross-sectional	72,817	U.K. Biobank adults	UPF % of total dietary intake	CRP	Higher UPF intake was associated with higher CRP after multivariable adjustment.
Lane M. [44]	2022	Australia	Full-text article	Cross-sectional	2018	Adults from the Melbourne Collaborative Cohort Study	Self-reported UPF intake	hs-CRP	Higher UPF intake was associated with higher hs-CRP.
Lopes A.E.S.C. [45]	2019	Brazil	Full-text article	Cross-sectional	8468	Civil servants from public universities/research institutions	UPF % of total energy intake	hs-CRP	Among women, the highest vs. lowest UPF tertile was associated with 14% higher mean CRP after adjustment for sociodemographics, smoking, and physical activity; among men, no association remained after sociodemographic adjustment.
Martins G.M.S. [46]	2022	Brazil	Full-text article	Cross-sectional	391	Adolescents	UPF % of total energy intake	hs-CRP, IL-6, TNF-α, IL-8, leptin	Higher UPF consumption was associated with increased hs-CRP, leptin, and IL-8 levels, while no significant associations were found for TNF-α or IL-6.
Millar S.R. [47]	2025	Ireland	Full-text article	Cross-sectional	1986	Primary-care adult patients	UPF % of total dietary intake	CRP, IL-6, TNF-α, PAI-1, leptin	Higher UPF intake was associated with higher CRP, IL-6, TNF-α and leptin, while PAI-1 was not significant.
Nestares T. [48]	2021	Spain	Full-text article	Cross-sectional	85 children (53 with celiac disease, 32 healthy controls)	Children with celiac disease on a gluten-free diet and healthy controls	UPF % of total dietary intake	IL-6, IL-1β, TNF-α, IL-8, MCP-1	No significant differences were found in TNF-α, IL-1β, IL-6, IL-8 or MCP-1 between celiac disease children consuming >50% of energy from UPF compared to those consuming <50% and to healthy controls.
Quetglas-Llabrés M.M. [49]	2023	Spain	Full-text article	Cross-sectional	92	Older adults with metabolic syndrome	UPF % of total dietary intake	IL-6, IL-1β, TNF-α, MCP-1, leptin	Higher UPF intake was associated with higher IL-6, TNF-α and leptin, while IL-1β and MCP-1 did not differ between UPF groups.
Silva Dos Santos F. [50]	2023	Brazil	Full-text article	Prospective cohort	524 (EPITeen, Portugal) + 2888 (Pelotas, Brazil)	Young adults from Porto (Portugal) and from Pelotas (Brazil)	UPF % of total energy intake	IL-6	Higher UPF consumption was associated with increased IL-6 concentrations among females in the EPITeen cohort and males in the Pelotas cohort.
Silva-Luis C.C. [51]	2024	Brazil	Full-text article	Cross-sectional	151	Children enrolled in public schools	UPF % of total energy intake	hs-CRP, IL-6, TNF-α	UPF consumption was not associated with hs-CRP, IL-6, or TNF-α levels among children.
Vivi A.C.P. [52]	2022	Brazil	Full-text article	Cross-sectional	90	Preterm infants from a hospital follow-up clinic and healthy term infants from primary care	UPF % of total energy intake (all types of milk excluded from the energy calculation)	CRP	Higher UPF consumption was associated with higher CRP, with a stronger association in preterm infants.
Xia L. [53]	2025	France	Full-text article	Cross-sectional	1594	Community adults	Self-reported UPF consumption	CRP	Total UPF consumption was not associated with CRP in fully adjusted models.
Zatsepina A. [54]	2025	U.S.	Conference proceeding	Retrospective cohort	796	Adults with histologically confirmed colon cancer	Self-reported dietary pattern	CRP, IL-6, TNF-α	Patients consuming high-UPF diets had elevated CRP, IL-6, and TNF-α compared to those on anti-inflammatory diets.
Zhao L. [55]	2024	U.S.	Full-text article	Prospective cohort	173,889	U.K. Biobank adults	UPF % of total dietary intake	CRP	Higher UPF intake was associated with increased odds of elevated CRP.
Zhao L. [56]	2023	U.S.	Full-text article	Cross-sectional	806 adolescents and 2734 adults	Community-dwelling participants from NHANES cohort	Self-reported UPF consumption	hs-CRP	In adults, higher UPF intake was associated with higher hs-CRP, whereas no association with hs-CRP was observed in adolescents.

Abbreviations: UPF, ultra-processed foods; CRP, C-reactive protein; hs-CRP, high-sensitivity C-reactive protein; IL, interleukin; TNF-α, tumor necrosis factor-α; MCP-1, monocyte chemoattractant protein-1; PAI-1, plasminogen activator inhibitor-1; U.S., United States; U.K., United Kingdom; NHANES, National Health and Nutrition Examination Survey.

**Table 2:** Biomarker-by-study summary of associations between ultra-processed food consumption and systemic inflammatory biomarkers in children/adolescents. A check mark (**✓**) denotes an association, whereas a cross (**X**) indicates no association.

First Author, Year	CRP	hs-CRP	IL-6	IL-1β	TNF-α	IL-8	MCP-1	PAI-1	Leptin
Bérard A., 2020 [35]	**X**		**✓**		**X**				**X**
Dos Santos G.R., 2025 [38]	**✓**								
Martins G.M.S., 2022 [46]		**✓**	**X**		**X**	**✓**			**✓**
Nestares T., 2021 [48]			**X**	**X**	**X**	**X**	**X**		
Silva-Luis C.C., 2024 [51]		**X**	**X**		**X**				
Vivi A.C.P., 2022 [52]	**✓**								
Zhao L., 2023 [56]		**X**							

**Table 3:** Biomarker-by-study summary of associations between ultra-processed food consumption and systemic inflammatory biomarkers in adults. A check mark (**✓**) denotes an association, whereas a cross (**X**) indicates no association.

First Author, Year	CRP	hs-CRP	IL-6	IL-1β	TNF-α	IL-8	MCP-1	PAI-1	Leptin
Bahrampour N., 2022 [33]		**✓**		**X**			**X**	**X**	
Baric L., 2025 [34]	**✓**								
Bérard A., 2020 [35]	**X**		**✓**		**X**				**X**
Contreras-Rodriguez O., 2023 [36]		**X**							
Cummings J., 2022 [37]	**✓**		**X**		**X**				
Hajmir M., 2023 [39]		**X**		**X**			**✓**	**✓**	
Hall K.D., 2019 [40]		**X**						**X**	**X**
Hosseininasab D., 2022 [41]		**✓**		**X**			**X**	**X**	
Kelsey P.T., 2022 [42]	**✓**								
Kityo A., 2025 [43]	**✓**								
Lane M., 2022 [44]		**✓**							
Lopes A., 2019 [45]	**X ** ✓ *								
Millar S.R., 2025 [47]	**✓**		**✓**		**✓**			**X**	**✓**
Quetglas-Llabrés M.M., 2023 [49]			**✓**	**X**	**✓**		**X**		**✓**
Silva dos Santos F., 2023 [50]			**✓**						
Xia L., 2025 [53]	**X**								
Zatsepina A., 2025 [54]	**✓**		**✓**		**✓**				
Zhao L., 2024 [55]	**✓**								
Zhao L., 2023 [56]		**✓**							

* Positive association between UPF consumption and CRP levels was found in women, but not in men.

## Data Availability

No new data were created or analyzed in this study. Data sharing is not applicable to this article.

## Supplementary Materials

The following supporting information can be downloaded at: https://www.mdpi.com/article/10.3390/nu17183012/s1.
