# Supplementary material for: Ultra-Processed Food Consumption and Systemic Inflammatory Biomarkers: A Scoping Review
Source: Nutrients. 2025 Sep 20;17(18):3012. doi: 10.3390/nu17183012 (PMC12472508; doi:10.3390/nu17183012)
Supplement: Supplementary file 1 [file nutrients-17-03012-s001.zip › Search strategy.pdf]

PubMed search string

("ultra-processed food"[tiab] OR "ultra processed food"[tiab] OR

ultraprocessed food[tiab] OR "ultra-processed foods"[tiab] OR

"ultra processed foods"[tiab] OR ultraprocessed foods[tiab])

AND

(C-Reactive Protein[Mesh] OR "C reactive protein"[tiab] OR "C-reactive protein"[tiab] OR

CRP[tiab] OR "high-sensitivity C-reactive protein"[tiab] OR "high sensitivity C reactive protein"[tiab] OR

hs-CRP[tiab] OR hsCRP[tiab] OR

Erythrocyte Sedimentation Rate[Mesh] OR erythrocyte sedimentation rate[tiab] OR ESR[tiab] OR

Fibrinogen[Mesh] OR fibrinogen[tiab] OR

Interleukin-6[Mesh] OR "interleukin 6"[tiab] OR "interleukin-6"[tiab] OR IL6[tiab] OR IL-6[tiab] OR

Interleukin-1beta[Mesh] OR "interleukin 1"[tiab] OR "interleukin-1"[tiab] OR

"interleukin-1 beta"[tiab] OR IL1[tiab] OR IL-1[tiab] OR IL-1β[tiab] OR IL-1b[tiab] OR

Tumor Necrosis Factor-alpha[Mesh] OR "tumor necrosis factor"[tiab] OR

TNF[tiab] OR TNF-alpha[tiab] OR TNFα[tiab] OR

Interleukin-8[Mesh] OR "interleukin 8"[tiab] OR "interleukin-8"[tiab] OR IL8[tiab] OR IL-8[tiab] OR

Chemokine CCL2[Mesh] OR chemokine CCL2[tiab] OR CCL2[tiab] OR MCP1[tiab] OR MCP-1[tiab] OR

Plasminogen Activator Inhibitor 1[Mesh] OR plasminogen activator inhibitor 1[tiab] OR PAI1[tiab] OR PAI-1[tiab] OR

Leptin[Mesh] OR leptin[tiab])

Web of Science search string

TS=("ultra-processed food" OR "ultra processed food" OR ultraprocessed food OR "ultra-processed foods" OR "ultra processed foods" OR ultraprocessed foods)

AND

TS=("C reactive protein" OR "C-reactive protein" OR CRP OR "high-sensitivity C-reactive protein" OR "high sensitivity C reactive protein" OR hs-CRP OR hsCRP OR

"erythrocyte sedimentation rate" OR ESR OR

fibrinogen OR

"interleukin 6" OR "interleukin-6" OR IL6 OR IL-6 OR

"interleukin 1 beta" OR "interleukin-1 beta" OR IL1 OR IL-1 OR "IL-1β" OR "IL-1b" OR

"tumor necrosis factor alpha" OR "tumor necrosis factor" OR TNF OR "TNF-alpha" OR "TNFα" OR

"interleukin 8" OR "interleukin-8" OR IL8 OR IL-8 OR

"chemokine CCL2" OR CCL2 OR MCP1 OR MCP-1 OR

"plasminogen activator inhibitor 1" OR PAI1 OR PAI-1 OR

leptin)

Embase search string

('ultra processed food':ti,ab OR 'ultra-processed food':ti,ab OR ultraprocessed:ti,ab OR 'ultra processed foods':ti,ab OR 'ultra-processed foods':ti,ab OR 'ultraprocessed foods':ti,ab)

AND

('c reactive protein'/exp OR 'c reactive protein':ti,ab OR crp:ti,ab OR 'high sensitivity c reactive protein':ti,ab OR 'high-sensitivity c reactive protein':ti,ab OR hs-crp:ti,ab OR hscrp:ti,ab OR

'erythrocyte sedimentation rate'/exp OR erythrocyte:ti,ab AND sedimentation:ti,ab AND rate:ti,ab OR esr:ti,ab OR

fibrinogen/exp OR fibrinogen:ti,ab OR

'interleukin 6'/exp OR 'interleukin 6':ti,ab OR il6:ti,ab OR il-6:ti,ab OR

'interleukin 1 beta'/exp OR 'interleukin 1 beta':ti,ab OR 'interleukin-1 beta':ti,ab OR il1:ti,ab OR il-1:ti,ab OR 'il-1β':ti,ab OR 'il-1b':ti,ab OR

'tumor necrosis factor alpha'/exp OR 'tumor necrosis factor alpha':ti,ab OR 'tumor necrosis factor':ti,ab OR tnf:ti,ab OR 'tnf-alpha':ti,ab OR 'tnfα':ti,ab OR

'interleukin 8'/exp OR 'interleukin 8':ti,ab OR 'interleukin-8':ti,ab OR il8:ti,ab OR il-8:ti,ab OR

'chemokine ccl2'/exp OR 'chemokine ccl2':ti,ab OR ccl2:ti,ab OR mcp1:ti,ab OR mcp-1:ti,ab OR

'plasminogen activator inhibitor 1'/exp OR 'plasminogen activator inhibitor 1':ti,ab OR pai1:ti,ab OR pai-1:ti,ab OR

leptin/exp OR leptin:ti,ab)
